# Unusual Cause of Vision Loss in a Case of Lupus

**DOI:** 10.31138/mjr.33.4.455

**Published:** 2022-12-31

**Authors:** Prasanta Padhan, Manmath Kumar Das, Amrita Pradhan, Debashis Maikap

**Affiliations:** 1 Department of Clinical Immunology and Rheumatology, Kalinga Institute of Medical Sciences, KIIT University, Bhubaneswar, Odisha, India,; 2 Department of Opthalmology, Kalinga Institute of Medical Sciences, KIIT University, Bhubaneswar, Odisha, India,; 3 Department of Ophthalmology, S.C.B Medical College, Cuttack, Odisha, India

**Keywords:** anaemia, retinopathy, Lupus

## Abstract

Lupus can affect vision in various ways, commonly due to vasculitic retinopathy, and can also result from ischemic optic neuropathy secondary to antiphospholipid syndrome. Anaemic retinopathy is most likely to occur in patients with severe anaemia or associated with thrombocytopenia. Causes of decreased visual acuity in anaemia include haemorrhages involving the macula, macular oedema, optic disc oedema and ischemic optic neuropathy. We herein describe an unusual cause of loss of vision in a lupus patient associated with severe anaemia and thrombocytopenia. Ocular examination was suggestive of anaemic retinopathy. Her vision improved with concomitant resolution of retinal findings in 1 month after treatment for lupus with anaemia correction. Hence, even though vision loss in lupus is dreadful, retinal changes in fundus examination should be carefully looked at to rule out anaemic retinopathy, as it is almost always reversible with correction of anaemia unlike lupus retinopathy.

## INTRODUCTION

Clinical presentation of lupus is variable, and any organ or system of body can be affected. Ocular manifestations in lupus can found up to one third of patients.^1^ The aetiology can be attributed to the immune complex deposition in the blood vessels of the conjunctiva, retina, choroid, sclera, ciliary body, also in the basement membranes of the ciliary body and cornea, and in the peripheral nerves of the ciliary body and conjunctiva leading to vasculitis and thrombosis.^2^ Lupus retinopathy is tissue microangiopathy, particularly associated with vasculitis or antiphospholipid antibodies, or both^3^; sometimes associated vision threatening complications secondary to optic nerve involvement and retinal vaso-occlusion. Usually, it correlates with systemic lupus disease activity.^2^ Anaemia has been reported as an important risk factor for developing retinopathy in many case series, with a prevalence of 20–28.3%.^4^ Anaemic retinopathy has been described in iron deficiency anaemia, aplastic anaemia, sickle cell anaemia, beta-thalassemia, pernicious anaemia, and drug-induced anaemia. However, anaemic retinopathy in lupus is rare and yet to be reported in the medical literature. We herein describe a patient with active lupus presented with loss of vision which was initially thought to be a case of lupus retinopathy, but turned out to be anaemic retinopathy on fundus examination.

## CASE REPORT

We report a case of lupus in a 40-year-old female who presented to us with sudden painless, non-progressive diminished vision in both eyes for 2 months. She had history of low-grade fever, easy fatigability, photosensitivity, malar rash of 3-month duration. She was frequently admitted elsewhere with history of multiple episodes of hematemesis and melena for last 3 months, for which she received multiple blood transfusions. On evaluation, she had severe thrombocytopenia (<20,000/cmm) detected on each admission and received intermittent oral prednisolone in suspicion of immune thrombocytopenic purpura. There was no prior history of ocular injury or head trauma. A clinical diagnosis of Systemic lupus erythematosus (SLE) was made. There was no history of joint pain, oral ulcer, muscle weakness, or Raynaud’s phenomenon. She had no other comorbidities such as diabetes mellitus or hypertension.

**Figure 1.**
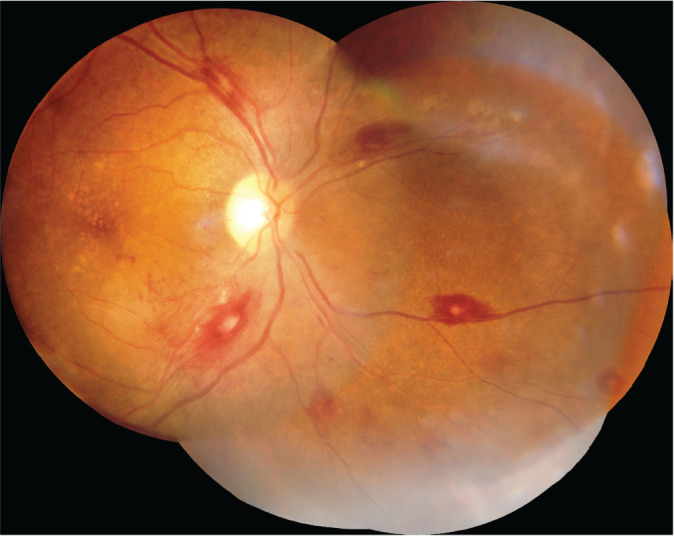
Right eye fundus photograph of posterior pole showing numerous flame-shaped haemorrhages, some with white centre (Roth spot) along with dot blot haemorrhages.

**Figure 2.**
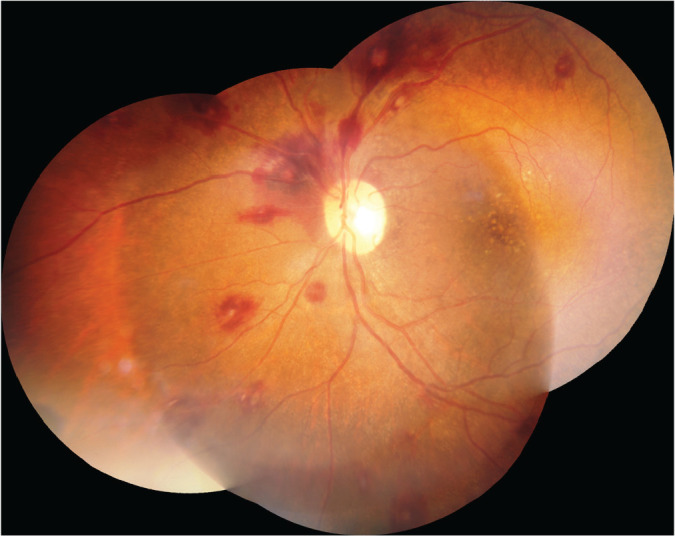
Left eye fundus photograph of posterior pole showing numerous flame-shaped superficial retinal haemorrhages, some with white centre (Roth spot) and peri-papillary subretinal haemorrhages.

On general examination, she had severe pallor with multiple petechial haemorrhages on oral mucosa and purpuric spots over bilateral lower limb. The best corrected visual acuity (BCVA) for far and near was 6/60 right eye (OD) and 6/36 left eye (OS). Pupillary responses and intraocular pressure were normal. Fundus examination showed numerous flame shaped haemorrhage with white centred (Roth spot) along with dot blot haemorrhage on posterior pole of right eye (**Figure 1**) and flame shaped superficial retinal haemorrhage some with white centred and peri-papillary subretinal haemorrhage (**Figure 2**) characteristic of anaemic retinopathy. Central nervous system examination and other systemic examinations were unremarkable.

Laboratory analysis showed C3 and C4 levels were reduced {64 mg/dL (reference range 90–180) and 4mg/dL (reference range 10–40) respectively}, ANA antinuclear antibody 4+ homogeneous and anti-DNA autoantibodies were positive (337 IU/mL). Work up for antiphospholipid syndrome was negative. Her serum Creatinine was 1.2 mg/dL, serum albumin 3.6 g/dL and proteinuria 250 mg in 24 hours. Her haemoglobin was 3.7g/dL (reference range 12.0–15.0g/dL), total leucocyte count 4200/cu mm (reference range 4,000–10,000/cu mm) and platelet count 3000/cu mm (reference range 150000–400000/cu mm). Her blood sugar (fasting and postprandial) was within normal range. Investigations for autoimmune haemolytic anaemia were negative. Total iron-binding capacity was 312 μg/dL (reference range 250–450) and ferritin was 56 ng/ml (reference range 18–270). Vitamin B12 was 628 pg/mL (reference range 200–835). Gastroscopy and colonoscopy revealed multiple petechial haemorrhages in mucosa. Contrast CT scan of the abdomen revealed no abnormalities to suggest gut vasculitis. MRI brain was unremarkable. Diagnosis of SLE with severe thrombocytopenia was made and she was started on pulse Methylprednisolone 1gm for 3 days followed by 1mg/kg Prednisolone. She had received 2 units of blood transfusion during hospital stay. Her counts improved and azathioprine was added as a steroid sparing agent. On follow up after one month, she had improved vision [(best corrected visual acuity (BCVA) for far and near was 6/9 right eye (OD) and 6/12 left eye (OS) with normal platelet counts (2.2 lakh/cu mm) and haemoglobin (12gm/dL)].

## DISCUSSION

The ocular changes found in anaemic retinopathy are nonspecific and may closely simulate diabetic or hypertensive retinopathy.^5^ Anaemic retinopathy usually affects both eyes; however, unilateral presentation is also reported. Anaemia can be due to nutritional deficiency like iron deficiency, vitamin B12, and folic acid deficiency. Other causes of anaemia include acute blood loss in menorrhagia, gastrointestinal haemorrhage, and chronic blood loss in parasitic disease. Transient retinal haemorrhages associated with anaemia from gastrointestinal haemorrhage were first described by Ulrich^1^ in 1883. Similarly, our case also had severe gastrointestinal blood loss due to severe thrombocytopenia which was improved on treatment of lupus.

Uncommon causes of anaemic retinopathy, such as inadequate production of erythrocytes (aplastic anaemia) or increased destruction of erythrocytes (haemolytic anaemia) do occur that may persist for a long time without any treatment. Secondary manifestation of other systemic diseases such as infection, malignancies like leukaemia, Waldenstrom’s Macroglobulinemia (WM,) and other autoimmune disorders can cause anaemia and subsequently anaemic retinopathy.

Retinopathy in patients with anaemia is a well known entity. Haemorrhages can be seen at all levels of the retina and choroid especially when thrombocytopenia coexists in diseases with severe anaemia such as leukaemia and aplastic anaemia.^6^

They may be most common type superficial and flame-shaped (located in the nerve fibre layer of the retina), dot or blot haemorrhages (in the inner retinal layers), or rarely preretinal or in the vitreous. Other common findings include Roth’s spots, exudates, cotton wool spots, retinal oedema, and venous tortuosity. Our patient had multiple superficial haemorrhages in retina associated with Roth’s spot. Roth’s spots or white centred haemorrhages are typically associated with bacterial endocarditis, and however, the association is not exclusive, since they occur in many other conditions including anaemia as in our case. The white centre could represent focal ischemia, inflammatory infiltrates, infectious organisms, fibrin and platelets, or an accumulation of neoplastic cells.^10^

The exact pathogenesis of anaemic retinopathy remains unclear and poorly understood. Factors such as anoxia, venous stasis, angiospasm, increased capillary permeability, and thrombocytopenia have been implicated in the pathogenesis of anaemic retinopathy.^7^ All retinal haemorrhages can occur when haemoglobin falls below 8 g/dL or if the platelet count falls below 50,000/cmm. As the severity of anaemia increases, the risk of retinopathy increases, particularly when the haemoglobin (Hb) level is below 6 g/dL.^9^ The combination of severe anaemia and thrombocytopenia is likely to produce retinal haemorrhages in the majority of patients (42–44%).^8,9^

Our patient also had severe anaemia (Hb-3.7g/L) and co-existent thrombocytopenia (platelet count 3000/cmm) at presentation. However, persistent anaemia and thrombocytopenia may have existed for a long time as her lupus was not diagnosed, and she had received multiple blood transfusions for the same at different occasions. The long-standing anaemia might be an “additional” factor in the causation of retinal haemorrhages through a prolonged damage of the vessel wall.

Lupus retinopathy is one of the most common causes of visually devastating consequences of systemic lupus erythematosus with an incidence of up to 29% in active lupus patients.^1^ Lupus retinopathy strongly correlated with occurrence of neurolupus.^11^ The earliest findings are small intraretinal haemorrhages and cotton wool spots. In addition, 5% to 10% of patients with SLE retinopathy in the presence of antiphospholipid antibodies will develop retinal vessel occlusion.^12^ Unlike anaemic retinopathy, here pathogenesis is attributed to deposition of immune complexes in the vessel wall and microemboli. Other autoimmune disease like vasculitis and antiphospholipid syndrome related retinopathy can also be associated with lupus. In this case the features were typical of anaemic retinopathy. Features which are classical of vasculitic retinopathy, such as perivascular sheathing, intraretinal infiltrates, cotton wool spots, and retinal necrosis were absent in our case.

Our patient’s vision improved rapidly within a week after blood transfusion because of improved retinal perfusion and eventually the retinal function which supported the diagnosis of anaemic retinopathy. On follow up after 1 month, she had resolution of haemorrhage and improvement of haemoglobin and platelet levels as a result of lupus treatment. This case highlights the fact that, even though sudden vision loss is alarming in a case of lupus, careful history taking with physical examination including fundus is warranted to avoid unnecessary investigations and aggressive immunosuppression.
